# Spread of *Cryptococcus gattii* in British Columbia, Canada, and Detection in the Pacific Northwest, USA

**DOI:** 10.3201/eid1301.060827

**Published:** 2007-01

**Authors:** Laura MacDougall, Sarah E. Kidd, Eleni Galanis, Sunny Mak, Mira J. Leslie, Paul R. Cieslak, James W. Kronstad, Muhammad G. Morshed, Karen H. Bartlett

**Affiliations:** * British Columbia Centre for Disease Control, Vancouver, British Columbia, Canada; † University of British Columbia, Vancouver, British Columbia, Canada; ‡ Washington State Department of Health, Shoreline, Washington, USA; § Oregon State Public Health, Portland, Oregon, USA

**Keywords:** Cryptococcus gattii, population surveillance, environmental exposure, British Columbia, detection, Pacific Northwest, research

## Abstract

Cryptococcus gattii, emergent on Vancouver Island, British Columbia (BC), Canada, in 1999, was detected during 2003–2005 in 3 persons and 8 animals that did not travel to Vancouver Island during the incubation period; positive environmental samples were detected in areas outside Vancouver Island. All clinical and environmental isolates found in BC were genotypically consistent with Vancouver Island strains. In addition, local acquisition was detected in 3 cats in Washington and 2 persons in Oregon. The molecular profiles of Oregon isolates differed from those found in BC and Washington. Although some microclimates of the Pacific Northwest are similar to those on Vancouver Island, C. gattii concentrations in off-island environments were typically lower, and human cases without Vancouver Island contact have not continued to occur. This suggests that C. gattii may not be permanently colonized in off-island locations.

In 1999, Cryptococcus gattii emerged on Vancouver Island, British Columbia (BC), Canada, among residents, visitors to the island, and domestic and wild animal populations. Disease incidence on Vancouver Island plateaued at 36 cases/million population/year during 2002–2005, markedly higher than rates reported in other C. gattii–endemic areas (*1**,**2*).

Unlike the closely related species C. neoformans, a common opportunistic pathogen of immunocompromised hosts, C. gattii affects primarily immunocompetent persons. Two C. gattii serotypes, B and C, have been described (*3*). The fungus is acquired through inhalation of airborne propagules and may cause pulmonary and central nervous system disease. Activities that disturb colonized soil or trees may increase the likelihood of exposure (*4*). Disease acquisition likely also depends on host factors, including underlying lung conditions and oral steroid use (M. Fyfe, unpub. data).

In a study of Vancouver Island human C. gattii serotype B cases from January 1999 through December 2001, infection was most common in men and those >60 years of age. Chest radiograph showed single or multiple pulmonary nodules in 68% of patients. Symptoms included severe cough and shortness of breath, often accompanied by chills, night sweats, and anorexia. Approximately 20% of patients had cryptococcal meningitis (M. Fyfe, unpub. data). The median incubation period was ≈6–7 months (*5*).

C. gattii has been isolated from more than 10 different native tree species on Vancouver Island and from the surrounding soil and air (*6**,**7*; Kidd et al., unpub. data). Despite sampling in areas both on and off the island, positive environmental isolates have, until recently, been confined to the Coastal Douglas Fir and very dry Coastal Western Hemlock biogeoclimatic zones along the east coast of Vancouver Island (*8*, Figure 1).

**Figure 1:**
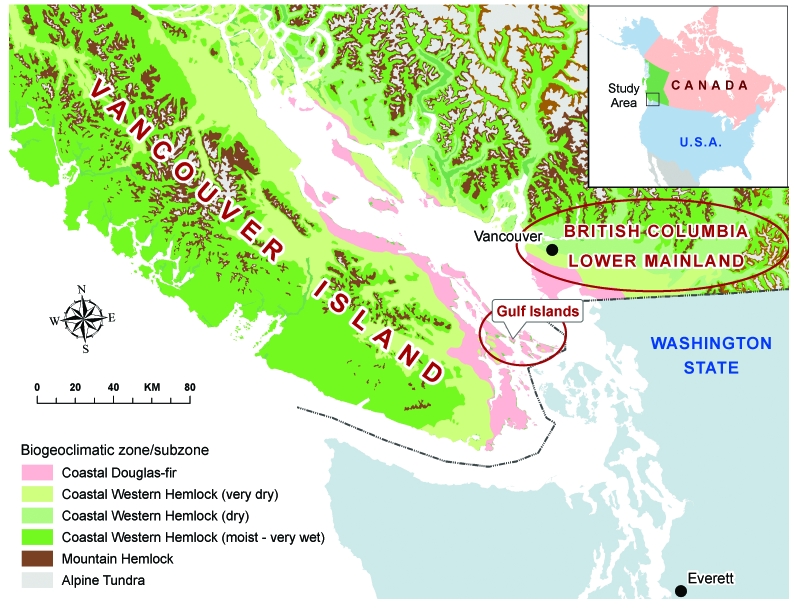
Biogeoclimatic and geopolitical boundaries within British Columbia.

VGIIa and VGIIb are the most commonly identified genotypes among human, animal, and environmental isolates from Vancouver Island (*6*). The VGI genotype has been isolated less frequently from clinical and environmental samples (*6**,**9*; Kidd et al., unpub. data).

The emergence of C. gattii infection on Vancouver Island, a temperate climate, was unusual because this species previously was associated only with tropical and subtropical climates (*10**,**11*). To facilitate surveillance activities, cryptococcal disease was made provincially notifiable in BC in 2003, formalizing laboratory reporting originally developed in response to disease emergence. A primary goal of surveillance was to monitor fungal spread to other areas of BC.

Vancouver Island is the largest island on the Pacific Coast of North America, covering 32,000 km^2^, with a population of ≈700,000. It is separated from the BC mainland by the Strait of Georgia, a body of water ≈50 km wide that contains several smaller islands known as the Gulf Islands (Figure 1). Travel among Vancouver Island, the BC mainland, and the Gulf Islands is very common, with an estimated 17.3 million passengers transported on BC Ferries' Vancouver Island routes annually (*12*).

Until 2004, all human cases of C. gattii infection reported to the British Columbia Centre for Disease Control were among those living on or traveling to Vancouver Island during the year before symptoms appeared. In December 2004, the first evidence of disease in humans without exposure to Vancouver Island or other known C. gattii–endemic areas was detected. This article summarizes the epidemiologic and environmental support for disease acquisition in parts of the BC lower mainland and focal areas of the US Pacific Northwest.

## Methods

### Human Surveillance

We interviewed persons from whom C. gattii serotype B was cultured through December 31, 2005, and who did not report contact with Vancouver Island or other known disease-endemic areas. We conducted telephone interviews by using a standard questionnaire to assess demographic information, travel history, risk factors for infection, underlying medical conditions, and clinical symptoms. Risk factors and travel exposures were assessed for the 1-year period before the onset of illness (or before diagnosis, in asymptomatic cases). Health authorities in neighboring provinces (Canada) and states (USA), where the disease is not reportable, were provided with case investigation forms and encouraged to investigate cryptococcal disease in immunocompetent persons.

### Animal Surveillance

Reports of animal cases were informally collected through veterinary networks in BC. Cases from the United States were reported by state veterinary epidemiologists. Infection in the animals was diagnosed histologically or identified as C. gattii serotype B by culture. None of the animals had traveled to Vancouver Island or other disease-endemic areas.

### Environmental Surveillance

From October 2001 through December 2005, environmental sampling was undertaken in the BC mainland, the BC Gulf Islands, and the US Pacific Northwest. We sampled 22 map grids as defined by the 1:50,000-scale National Topographic System of Canada (NTS) and US Geological Survey (USGS) mapping system. Geographic data were assembled as described elsewhere (Kidd et al., unpub. data). Purposive sampling was conducted at selected sites and areas surrounding the homes of persons with C. gattii infection, those who reported travel to Vancouver Island and those who did not. Sampled environments included front and back yards, walking trails, public parks, and recreational areas. Trees, small woody debris, soil, air, and water were sampled as described elsewhere (Kidd et al., unpub. data).

Sample positivity was scored binarily. C. gattii concentration was expressed as CFU/gram, CFU/m^3^, and CFU/100 mL in soil, air, and water, respectively. The concentrations of multiple samples were described by the geometric mean and geometric standard deviation. When more than 1 sample was taken from a single sampling point (e.g., the same tree), only the first sample was included.

### Identification and Genetic Characterization

We initially cultured the samples on Staib media (*13*). Resulting dark brown colonies were grown on canavanine-glycine-bromothymol blue (CGB) agar (*14*) to differentiate C. gattii from C. neoformans and then serotyped (Crypto-check, Iatron Laboratories, Tokyo, Japan).

Molecular types were identified by a previously described PCR-based restriction fragment length polymorphism (RFLP) method (*15*), which was adapted for further discrimination of variation within the VGII molecular type (*9*). The URA5 gene was amplified as previously described (*15*) and then completely digested at 37°C in a 20-μL reaction containing 1× NEB2 buffer, 1× bovine serum albumin, and 4 U each of Hha I, Dde I, and BsrG I (New England Biolabs, Inc., Ipswich, MA, USA). RFLP products were subjected to electrophoresis and visualized on a 3% agarose gel prestained with ethidium bromide. Control strains were used for each possible C. gattii RFLP pattern: WM179 (VGI), NIH444 (VGIIa), RB28 (VGIIb), WM161 (VGIII), and WM779 (VGIV). C. neoformans strains WM148 (VNI), WM626 (VNII), WM628 (VNIII), and WM629 (VNIV) were also included.

Multilocus sequence typing was performed for selected isolates by using methods previously described (*8*) with the use of 2 additional loci, PLB1 and IGS (*16*). We isolated total DNA from histopathology specimens (n = 3) by using the DNeasy Tissue kit (QIAGEN Inc., Mississauga, Ontario, Canada). Cryptococcal-specific PCR-RFLP was conducted as described above. The internal transcribed spacer region (ITS1-5.8S-ITS2) was amplified and sequenced for identification of C. gattii–specific polymorphisms (*17*).

## Results

### Epidemiology of Human Infection

**Figure 2:**
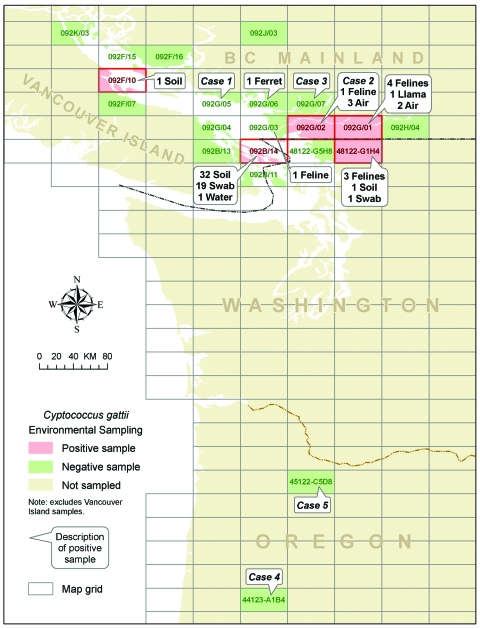
Location of human and animal Cryptococcus gattii cases and positive environmental samples found off Vancouver Island.

Five persons with culture-confirmed C. gattii, 3 in BC and 2 in Oregon, did not report exposure to Vancouver Island or other cryptococcal disease–endemic areas (Figure 2). Case-patients 1 through 4 received a diagnosis or reported symptom onset from September through December 2004. Case-patient 5, who had a fatal infection, received a diagnosis in December 2005.

Case-patient 1 was a 47-year-old man living in BC who was hospitalized with cough, chills, night sweats, nausea, loss of appetite, muscle pain, headache, and neck stiffness. Both lung and brain cryptococcomas were identified. He had chronic hepatitis C infection and a history of drug addiction. At the time of infection, he smoked 20–40 cigarettes/day. His residence, a farmhouse undergoing significant renovations, was located in NTS grid 092G/05 on the coast north of Vancouver. Environmental exposures included yard and landscaping work at this property.

Case-patient 2 was a 48-year-old woman living in BC who experienced shortness of breath, fever, chills, headache, night sweats, loss of appetite, nausea, and muscle pain. A lung mass was identified by computed tomography. She had no known underlying health conditions. She resided on the BC lower mainland within NTS grid 092G/02; her last visit to Vancouver Island was 4 years before the onset of her illness. In the year before onset, considerable deforestation had occurred near her residence to clear land for housing developments. During this period, she also traveled ≈1 day/week to garden centers and nurseries within NTS 092G/02 to obtain shrubs, trees, and new topsoil for yard landscaping she carried out at her residence.

Case-patient 3 was a 73-year-old woman living in BC who had chronic renal failure requiring dialysis and a history of lung disease and breast cancer. She was asymptomatic; a cryptococcal lung nodule was identified radiographically after she had hip surgery in December 2004. No nodule was apparent on imaging conducted <2 months earlier, which suggests recent acquisition. The patient resided in NTS grid 092G/07. She last visited Vancouver Island 14 years before her diagnosis. She had reduced mobility and consequently little outdoor exposure.

Case-patient 4 was a 59-year-old man living in Oregon who began to experience cough, shortness of breath, fever, chills, weight loss, nausea, and muscle pain in December 2004. He had undergone a kidney transplant in September 2003 and reported scarring of lung tissue due to his occupation. His place of residence was located within USGS grid 44123-A1B4. He had not traveled outside Oregon in the year before symptom onset.

Case-patient 5 was an 87-year-old man living in Oregon (USGS grid 45122-C5D8). He was hospitalized in December 2005 with meningitis, accompanied by fever, weight loss, and loss of appetite. His medical history included chronic lymphocytic leukemia, and he had taken oral steroids in the year before diagnosis. He had traveled to parts of Oregon, Washington, and Colorado during his exposure period.

### Epidemiology of Off-island Animal Cases

In BC, a retrospective review of companion animal cases identified 8 culture-confirmed serotype B cases, which occurred in a ferret, a llama, and 6 cats. Specimens were collected from December 2003 through December 31, 2005; animal residences were located throughout the BC lower mainland (Figure 2).

In Washington, 3 cats with cryptococcal disease residing in USGS 48122-G1H4, close to the BC-USA border, were reported from February through June 2005 (Figure 2). All cases were diagnosed by histopathologic examination, and no cultures were obtained.

### Environmental Sampling

From October 2001 through December 2005, 3% of 2,033 off-island environmental samples were positive for C. gattii (Table 1). Swab samples included 45% of samples from trees and other structures (n = 925), 38% from soil (n = 781), 15% from air (n = 304), and 1% from water (n = 23).

**Table 1:** Summary of sampling results from locations off Vancouver Island*

Sample type	BC mainland	Gulf Islands	Washington, USA	Oregon, USA	Total
Air	196	91	11	6	304
Negative	191	91	11	6	299
Positive (%)	5 (3)	0	0	0	5 (2)
Soil	408	250	28	95	781
Negative	408	217	27	95	747
Positive (%)	0	33 (13)	1 (4)	0	34 (4)
Swab	521	272	38	94	925
Negative	521	253	37	94	905
Positive (%)	0	19 (7)	1 (3)	0	20 (2)
Water	15	6	–	2	23
Negative	15	5	–	2	22
Positive (%)	0	1 (17)	–	0	1 (4)
Total	1,140	619	77	197	2,033
Negative	1,135	566	75	197	1,973
Positive (%)	5 (0)	53 (9)	2 (3)	0	60 (3)

*BC, British Columbia.

**Figure 3:**
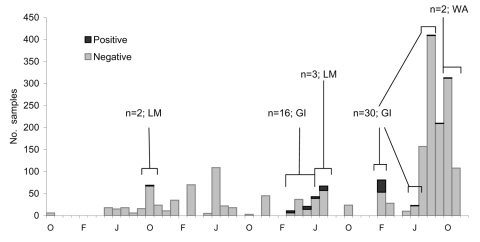
Summary of non–Vancouver Island environmental sampling effort, October 2001–December 2005. LM, lower mainland; GI, Gulf Islands; WA, Washington.

Five positive air samples were recovered from 2 focal areas of the lower mainland at 2 different times (Figure 3). The first 2 were collected from air in NTS grid 092G/02 on the same day in October 2002. No other positive samples were detected in 2003 despite further sampling at this site and others. In July 2004, a third positive air sample was collected from grid 092G/02 and 2 more from grid 092G/01. Despite extensive sampling in these areas and other parts of the BC lower mainland (n = 1,140), C. gattii was not isolated from colonized sources such as trees or soil.

Among environmental samples taken outside Vancouver Island, C. gattii was most often recovered from the Gulf Islands (Table 1). NTS grid 092B/14 had the highest proportion of positive samples (52/220; 24%). Colonized sources were identified from a single Gulf Island within this grid in March, May, and June of 2004. In 2005, positive soil and tree samples were obtained in February, August, September, and October from 3 of the Gulf Islands (NTS grids 092B/14 and 092F/01) and Washington (USGS grid 48122-G1H4). From among 50 samples collected a month apart in Washington within 10 km of the BC border (USGS grid 48122-G1H4), a single soil sample and a swab of a fence post were positive. An additional 27 samples from other sites along the US side of the border (USGS grids 48122-G1H4 and 48122-G5H8) were negative, as were all 197 samples collected from several areas of Oregon (USGS grids 44123-A1B4 and 45122-C5D8). The geometric mean concentration of detected C. gattii among soil, water, and air samples taken from various sites outside Vancouver Island is summarized in Table 2.

**Table 2:** Detected concentration of *Cryptococcus gattii* in positive soil and air samples from British Columbia and Washington*

Concentration of *C. gattii*	Vancouver Island	BC mainland	Disease-endemic Gulf Island	Other Gulf Islands	Washington, USA
In soil (CFU/g)					
N	143	0	31	2	1
Geometric mean	193.7	–	632	33.9	70.8
Geometric SD	6.5	–	14.2	1.5	–
Range	10–36,350	–	24–192,952	24.8–45.5	–
In air (CFU/m^3^) for comparable months of the year					
N	24	5	0	0	0
Geometric mean	43.3	10.8	–	–	–
Geometric SD	4.6	2.7	–	–	–
Range	2–875	5–38	–	–	–

*BC, British Columia; SD, standard deviation.

### Molecular Typing Results for Human, Animal, and Environmental Isolates

**Figure 4:**
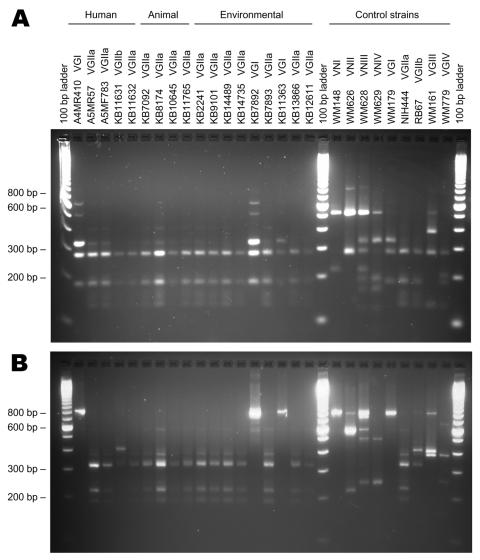
URA5–restriction fragment length polymorphism (RFLP) profiles for selected human, animal, and environmental Cryptococcus gattii isolates. A) URA5-RFLP to determine the molecular type using Hha I and Sau96 I endonucleases (14). B) URA5-RFLP to confirm molecular type and determine VGII subtype, using Hha I, Dde I, and BsrG I endonucleases.

Table 3 and Figure 4 summarize the molecular subtyping results for human and animal isolates from persons and animals with no recent exposure to Vancouver Island, as well as environmental isolates obtained from off-island locations.

**Table 3:** Geographic location and molecular type associated with clinical and environmental isolates from locations off Vancouver Island*

Isolate	Date†	Host	Residence	Geographic grid	Culture/specimen no.	Serotype	Molecular type
Human							
1	Dec 2004	Human	BC mainland	NTS 092G/05	A4MR410	B	VGI
2	Mar 2005	Human	BC mainland	NTS 092G/07	A5MF738	B	VGIIa
3	Mar 2005	Human	BC mainland	NTS 092G/02	A5MR57	B	VGIIa
4	2005‡	Human	Oregon	USGS 44123-A1B4	KB11632	B	VGIIa§
5	Dec 2005	Human	Oregon	USGS 45122-C5D8	A6MR38	B	VGIIb§
Animal							
1	Nov 2003	Llama	BC mainland	NTS 092G/01	KB7092	B	VGIIa
2	Mar 2004	Cat	BC mainland	NTS 092G/01	KB8174	B	VGIIa
3	May 2004	Cat	BC mainland	NTS 092G/01	KB8686	B	VGIIa
4	Aug 2004	Cat	BC mainland	NTS 092G/01	KB10645	B	VGIIa
5	Nov 2004	Cat	BC mainland	NTS 092G/01	KB11242	B	VGIIa
6	Mar 2005	Cat	BC mainland	NTS 092G/03	KB11765	B	VGIIa
7	Sep 2005	Ferret	BC mainland	NTS 092G/06	KB14724	B	VGIIa
8	2005‡	Cat	BC mainland	NTS 092G/01	KB15181	B	VGIIa
9	Jul 2004	Cat	Washington	USGS 48122-G1H4	2004-7975¶	B	VGIIa
10	Jan 2005	Cat	Washington	USGS 48122-G1H4	2005-0550¶	B	VGIIa
11	Apr 2005	Cat	Washington	USGS 48122-G1H4	2005-4659¶	B	VGIIa
Representative environmental isolates (of 60 total)							
–	Oct 2002	Air	BC mainland	NTS 092G/02	KB2045	B	VGIIa
–	Oct 2002	Air	BC mainland	NTS 092G/02	KB2241	B	VGIIa
–	Jul 2004	Air	BC mainland	NTS 092G/02	KB9057	B	VGIIa
–	Jul 2004	Air	BC mainland	NTS 092G/01	KB9101	B	VGIIa
–	Jul 2004	Air	BC mainland	NTS 092G/01	KB9091#	B	–
–	Mar 2004	Swab, tree	Gulf Islands	092B/14	KB7892	B	VGI
–	Mar 2004	Soil	Gulf Islands	092B/14	KB7893	B	VGIIa
–	Feb 2005	Swab, tree	Gulf Islands	092B/14	KB11363	B	VGI
–	Jun 2005	Soil	Gulf Islands	092B/10	KB12611	B	VGIIa
–	Aug 2005	Soil	Gulf Islands	092B/14	KB13866	B	VGIIa
–	Sep 2005	Swab, fence post	Washington	USGS 48122-G1H4	KB14489	B	VGIIa
–	Oct 2005	Soil	Washington	USGS 48122-G1H4	KB14735	B	VGIIa

*BC, British Columbia; NTS, National Topographic System of Canada; USGS, US Geological Survey. †Date of diagnosis for human and animal case-patients; date of sample for environmental isolates. ‡Month unknown. §Multilocus sequence typing analyses showed differences between these isolates and characterized VGIIa and VGIIb strains from British Columbia. ¶DNA isolated from formalin-fixed, paraffin-embedded tissue. #Isolate could not be cleaned from contaminants. Not retained.

Of the 5 human cases, 3 were attributed to the VGIIa molecular type, 1 to the VGIIb molecular type, and 1 to the VGI molecular type. However, although all 3 molecular types have been identified among clinical and environmental isolates from Vancouver Island, multilocus sequence typing (MLST) results indicated that both the VGIIa and VGIIb strains from Oregon cases were genetically distinct from previously characterized Vancouver Island isolates (*9**,**16*). The Oregon case 4 VGIIa isolate differed from Vancouver Island VGIIa at 1 locus, while Oregon case 5 VGIIb differed from Vancouver Island isolates at 4–5 loci, where it was more similar to Vancouver Island VGIIa than VGIIb (Table 4).

**Table 4:** Multilocus sequence typing (MLST) profiles of representative VGII strains from Vancouver Island compared with *Cryptococcus gattii* isolates from clinical and environmental sources in other locations*

Culture no.	Origin	Source	RFLP genotype	MLST profiles					
				URA5	LAC	FTR1	CAP1	PLB1	IGS
A1M R265	VI	Human	VGIIa	5†	3†	4†	2†	1†	1†
A1M R272	VI	Human	VGIIb	7‡	3	4	3‡	2‡	2‡
A5M R57	LM	Human	VGIIa	5	3	4	2	1	1
A5M F738	LM	Human	VGIIa	5	3	4	2	1	1
KB7092	LM	Animal	VGIIa	5	3	4	2	1	1
KB11765	LM	Animal	VGIIa	5	3	4	2	1	1
KB2045	LM	Air	VGIIa	5	3	4	2	1	1
KB13866	GI	Soil	VGIIa	5	3	4	2	1	1
KB11377	GI	Soil	VGIIa	5	3	4	2	1	1
KB14489	WA	Fence post	VGIIa	5	3	4	2	1	1
KB14735	WA	Soil	VGIIa	5	3	4	2	1	1
KB11632	OR	Human	VGIIa	12§	3	4	2	1	1
A6M R38	OR	Human	VGIIb	11¶	3	9¶	4#	1	3¶

*RFLP, restriction fragment length polymorphism; VI, Vancouver Island; LM, British Columbia lower mainland; GI, Gulf Islands; WA, northern Washington, USA; OR, Oregon, USA. †Representative sequence accession nos.: URA5 = AY973136, LAC = AY973094, FTR1 = AY972024, CAP1 = AY971991, PLB1 = DQ777861, IGS = DQ777859. ‡ Representative sequence accession nos.: URA5 = AY973141, CAP1 = AY971981, PLB1 = DQ777862, IGS = DQ777860. §Accession no. for unique sequences: URA5 = DQ777864. ¶Accession no. for unique sequences: URA5 = DQ777863, FTR1 DQ777857, IGS DQ777858. #Representative sequence accession no.: CAP1 = AY971973.

Cryptococcal DNA isolated from the formalin-fixed, paraffin-embedded tissue of 3 cats in Washington belonged to the VGIIa molecular type. MLST profiles could not be determined in these cases because of the relatively poor quality and yield of DNA from the fixed tissue.

Most off-island environmental isolates that were typed belonged to the VGIIa molecular type. These included 4 of the 5 isolates from lower mainland air samples (the fifth could not be separated from contaminants) and 90% of 20 typed isolates from the NTS grid with the highest proportion of positive off-island environmental samples (092B/14). All tested environmental VGIIa isolates from BC and Washington possessed identical MLST profiles to those of representative isolates from Vancouver Island (Table 3).

## Discussion

Surveillance for C. gattii, conducted in BC since the pathogen emerged on Vancouver Island in 2001, identified its spread to off-island locations in 2005. To date, 3 humans and 8 animals residing within the BC lower mainland who had not traveled to Vancouver Island or other known cryptococcal disease–endemic areas within the incubation period for disease have been found to have culture-confirmed C. gattii infection. All but 1 case belonged to the VGIIa subtype, the dominant genotype among clinical and environmental isolates from Vancouver Island (*6**,**9**,**16*). Human surveillance findings are supported by a parallel spread of C. gattii to animal populations on the BC mainland and positive air samples in this area. In addition, C. gattii infections with no recent link to Vancouver Island or other disease-endemic areas have been reported in 3 cats in Washington and 2 persons in Oregon. These cases represent the first evidence of local disease acquisition in this part of the United States. One historic case of VGIIa C. gattii (NIH444) was diagnosed in Seattle in the early 1970s; however, residence and travel history for the infected person are unknown (*6**,**9**,**16*).

Before the identification of new disease–endemic areas, all humans with C. gattii infection either lived within or traveled to the Coastal Douglas Fir and very dry Coastal Western Hemlock biogeoclimatic zones (Figure 1), located along the eastern edge of Vancouver Island. These zones are characterized by warm, dry summers and mild, wet winters and extend into the southern Gulf Islands and the BC lower mainland. Climates with comparable rainfall and temperature extend south into parts of Washington and Oregon in the United States (*18*). Franklin and Dyrness (*19*) identified plant communities similar to those in BC in the San Juan Islands and Puget Trough of Washington and the Willamette Valley in Oregon. These ecologic likenesses to BC support the idea that C. gattii may colonize niche areas of the US Pacific Northwest.

Although microclimate similarities exist, we could not determine whether the isolation of C. gattii from areas outside Vancouver Island represents true colonization or transient dispersal of the organism at the time of sampling, such as through wind flow or mechanical vectors/fomites. Despite repeated sampling, no environmental source (e.g., tree, soil) of the VGIIa isolates detected in air on the BC mainland has been found. Either an undiscovered reservoir exists on the BC mainland or detectable airborne C. gattii was aerosolized and dispersed from known colonized sources, such as Vancouver Island or the Gulf Islands. Washington VGIIa environmental isolates, identical by MLST to those from BC, may represent recent dispersal from BC or independent foci of colonization.

Sampling sites on Vancouver Island have shown different patterns of C. gattii colonization over time (*4*). Transiently positive sites are characterized by a positive C. gattii isolation, followed by a series of negative samples over a period of months or years. Permanently colonized sites consistently yield positive samples. Intermittently colonized sites yield cycles of positive and negative samples over time, perhaps the result of population fluctuation above and below the limits of detection as the organism competes with local microbiota, while it adjusts to a new ecologic niche. Repeated sampling of non–Vancouver Island sites previously positive for C. gattii may show the extent of colonization and the likelihood of these areas becoming C. gattii–endemic.

The detected concentration of C. gattii in air and soil samples from the BC lower mainland and northern Washington was lower than in samples from Vancouver Island. Based on a comparison of geometric means, the detected C. gattii concentration in air samples from the mainland was 4-fold lower than in Vancouver Island air samples collected at the same time of the year (Table 2). C. gattii concentration in soil from northern Washington and 2 of the Gulf Islands was ≈5× lower than in soil from Vancouver Island. Only in a limited area of 1 particular Gulf Island in grid 092B/14 was C. gattii concentration in soil higher (3.3-fold) than in soil from Vancouver Island.

While direct comparisons with infected persons living on or traveling to Vancouver Island are not possible because of the small number of off-island cases, humans affected by C. gattii in off-island environments may have a higher rate of serious underlying health conditions. Among cases in BC and Oregon, persons were affected by renal failure, chronic hepatitis C infection, and cancer (i.e., chronic lymphocytic leukemia, breast cancer). In an age-matched case-control study, persons from Vancouver Island with C. gattii infection were not significantly more likely than noninfected island residents to have had cancer (M. Fyfe, unpub. data) or liver disease (L. MacDougall, unpub. data). Persons with compromised immune systems may be more susceptible to infection with C. gattii at the lower concentrations observed in most off-island environments.

Even with ongoing surveillance in BC, the last reported case of symptom onset in a person with C. gattii infection who had not traveled to Vancouver Island was in December 2004. As of May 2006, no further cases had been detected, a finding at odds with the notion of permanent colonization. The onset of infections in the lower mainland of BC (September–December 2004) coincides with positive air samples on the mainland some months earlier (July 2004), given known variations in individual incubation periods (Figure 3) (*5*). Although animal cases did continue to occur during 2005–2006, environmental sampling attempts throughout 2005 did not detect the organism in the lower mainland. This result indicates either that permanent colonization did not occur in the sampled areas or that C. gattii was present below the limits of detection.

As on Vancouver Island, C. gattii in off-island areas was first detected in companion animals. Animal cases began to be regularly identified in March 2004, ≈6 months before human illness was reported in this area. As on Vancouver Island and in Australia, cats were affected more often than other companion animals (*20**,**21*). However, illness occurred in a ferret and llama, rare animals also infected early in the emergence on Vancouver Island, which may suggest that these species are particularly sensitive to infection. Despite substantial underreporting of animal cases, data from Vancouver Island suggest that animal cases exceeded human cases by almost 75%, highlighting their value as a sentinel indicator of disease (*20*).

Clinical and environmental isolates from the BC mainland, Gulf Islands, and northern Washington tested by MLST were identical to representative isolates from Vancouver Island at the 6 loci investigated (Table 4) (*9**,**16*). However, although isolates from the C. gattii human case-patients living in Oregon were typed as VGIIa and VGIIb, MLST analyses indicated that these isolates were genetically distinct from BC and Washington clinical and environmental isolates. A BLAST comparison (http://www.ncbi.nlm.gov/BLAST) to sequences from previous studies and those represented in the National Center for Biotechnology Information database identified Oregon strains as genotypically unique (*6**,**16*). We have not identified an environmental source of C. gattii within Oregon or any isolate possessing the same MLST profile as the Oregon clinical strains. Oregon strains could represent an independent population; alternatively, they may have evolved from the VGIIa or VGIIb strains previously described in BC or from VGIIa strains from California (*6**,**9**,**16*), either through random genetic drift or through sexual recombination. Recent studies suggest that same-sex mating can occur among cryptococcal isolates and that the VGIIa genotype may have arisen from same-sex mating between a strain of the VGIIb genotype and another unknown strain (*16**,**22*).

## Conclusion

C. gattii infections have been shown in human and animal residents of the BC lower mainland and in Washington and Oregon in the United States, despite no contact with Vancouver Island or other known disease-endemic areas. These findings may represent an expansion of recognized areas where the disease is endemic.

## References

[1] Chen S, Sorrell T, Nimmo G, Speed B, Currie B, Ellis D, MedlineEpidemiology and host- and variety-dependent characteristics of infection due to Cryptococcus neoformans in Australia and New Zealand. Australasian Cryptococcal Study Group. Clin Infect Dis. 2000;31:499–508. 10.1086/313992 10987712

[2] Sorrell TC. MedlineCryptococcus neoformans variety gattii. Med Mycol. 2001;39:155–68. 10.1080/714031012 11346263

[3] Bennett JE, Kwon-Chung KJ, Howard DH. MedlineEpidemiologic differences among serotypes of Cryptococcus neoformans. Am J Epidemiol. 1977;105:582–6. 326036 10.1093/oxfordjournals.aje.a112423

[4] Kidd SE, Bach PJ, Hingston AO, Mak S, Chow Y, MacDougall L, Dispersal of Cryptococcus gattii, British Columbia, Canada. Emerg Infect Dis [serial on the Internet]. 2007 Jan [date cited]. Available from http://www.cdc.gov/ncidod/EID/13/1/42.htm 10.3201/eid1301.060823 PMC2725814 17370515

[5] MacDougall L, Fyfe M. MedlineEmergence of Cryptococcus gattii in a novel environment provides clues to its incubation period. J Clin Microbiol. 2006;44:1851–2. 10.1128/JCM.44.5.1851-1852.2006 16672420 PMC1479218

[6] Kidd SE, Hagen F, Tscharke RL, Huynh M, Bartlett KH, Fyfe M, MedlineA rare genotype of Cryptococcus gattii caused the cryptococcosis outbreak on Vancouver Island (British Columbia, Canada). Proc Natl Acad Sci U S A. 2004;101:17258–63. 10.1073/pnas.0402981101 15572442 PMC535360

[7] Bartlett K, MacDougall L, Mak S, Duncan C, Kidd S, Fyfe M. Cryptococcus gattii: a tropical pathogen emerging in a temperate climate zone. Proceedings 16th Conference on Biometeorology and Aerobiology; 2004 Aug 25–26; Vancouver, British Columbia, Canada. Boston: American Meterological Society. Abstract no. 5.5.

[8] Biogeoclimatic Zones of British Columbia. Victoria (BC); 2001. Government of British Columbia, Ministry of Forests. [cited 22 Jun 2006]. Available from http://www.for.gov.bc.ca/hfd/library/documents/treebook/biogeo/biogeo.htm

[9] Kidd SE, Guo H, Bartlett KH, Xu J, Kronstad JW. MedlineComparative gene genealogies indicate that two clonal lineages of Cryptococcus gattii in British Columbia resemble strains from other geographical areas. Eukaryot Cell. 2005;4:1629–38. 10.1128/EC.4.10.1629-1638.2005 16215170 PMC1265896

[10] Kwon-Chung KJ, Bennett JE. MedlineHigh prevalence of Cryptococcus neoformans var. gattii in tropical and subtropical regions. Zentralbl Bakteriol Mikrobiol Hyg [A]. 1984;257:213–8. 6207684

[11] Kwon-Chung KJ, Bennett JE. MedlineEpidemiologic differences between the two varieties of Cryptococcus neoformans. Am J Epidemiol. 1984;120:123–30. 6377880 10.1093/oxfordjournals.aje.a113861

[12] Regional Profile. Vancouver Island, Victoria and the Gulf Islands. Tourism British Columbia; 2005. [cited 22 Jun 2006]. Available at: http://www.tourismbc.com/PDF/RegionalProfile_TAVancouver Island_FINAL.pdf

[13] Staib F, Seibold M, Antweiler E, Frohlich B, Weber S, Blisse A. MedlineThe brown colour effect (BCE) of Cryptococcus neoformans in the diagnosis, control and epidemiology of C. neoformans infections in AIDS patients. Zentralbl Bakteriol Mikrobiol Hyg [A]. 1987;266:167–77. 3321763 10.1016/s0176-6724(87)80030-5

[14] Kwon-Chung KJ, Polacheck I, Bennett JE. MedlineImproved diagnostic medium for separation of Cryptococcus neoformans var. neoformans (serotypes A and D) and Cryptococcus neoformans var. gattii (serotypes B and C). J Clin Microbiol. 1982;15:535–7. 7042750 10.1128/jcm.15.3.535-537.1982 PMC272134

[15] Meyer W, Castaneda A, Jackson S, Huynh M, Castaneda E. MedlineMolecular typing of IberoAmerican Cryptococcus neoformans isolates. Emerg Infect Dis. 2003;9:189–95. 12603989 10.3201/eid0902.020246 PMC2901947

[16] Fraser JA, Giles SS, Wenink EC, Geunes-Boyer SG, Wright JR, Diezmann S, MedlineSame-sex mating and the origin of the Vancouver Island Cryptococcus gattii outbreak. Nature. 2005;437:1360–4. 10.1038/nature04220 16222245

[17] Katsu M, Kidd S, Ando A, Moretti-Branchini ML, Mikami Y, Nishimura K, MedlineThe internal transcribed spacers and 5.8S rRNA gene show extensive diversity among isolates of the Cryptococcus neoformans species complex. FEMS Yeast Res. 2004;4:377–88. 10.1016/S1567-1356(03)00176-4 14734018

[18] Meidinger D, Pojar J. Ecosystems of British Columbia. Special report series no. 6. Victoria (BC): Ministry of Forests; 1991.

[19] Franklin JF, Dyrness CT. Natural vegetation of Oregon and Washington. General technical report, no. PNW-8. Portland (OR): Department of Agriculture and Forest Services; 1973.

[20] Duncan C. The emergence of Cryptococcus gattii in British Columbia: veterinary aspects. [MSc dissertation]. Saskatoon, Saskatchewan, Canada: University of Saskatoon; June 2005.

[21] O'Brien CR, Krockenberger MB, Wigney DI, Martin P, Malik R. MedlineRetrospective study of feline and canine cryptococcosis in Australia from 1981 to 2001: 195 cases. Med Mycol. 2004;42:449–60. 10.1080/13693780310001624547 15552647

[22] Lin X, Hull CM, Heitman J. MedlineSexual reproduction between partners of the same mating type in Cryptococcus neoformans. Nature. 2005;434:1017–21. 10.1038/nature03448 15846346

